# Qianliening capsule inhibits benign prostatic hyperplasia angiogenesis via the HIF-1α signaling pathway

**DOI:** 10.3892/etm.2014.1723

**Published:** 2014-05-19

**Authors:** JIUMAO LIN, JIANHENG ZHOU, WEI XU, ZHENFENG HONG, JUN PENG

**Affiliations:** 1 Academy of Integrative Medicine Biomedical Research Center, Fujian University of Traditional Chinese Medicine, Fuzhou, Fujian 350122, P.R. China; 2 Fujian Key Laboratory of Integrative Medicine on Geriatrics, Fujian University of Traditional Chinese Medicine, Fuzhou, Fujian 350122, P.R. China; 3 Department of Integrative Medicine, Fujian University of Traditional Chinese Medicine, Fuzhou, Fujian 350122, P.R. China; 4 Department of Pharmacology, Fujian University of Traditional Chinese Medicine, Fuzhou, Fujian 350122, P.R. China

**Keywords:** qianliening capsule, benign prostatic hyperplasia, angiogenesis, hypoxia-inducible factor-1α, vascular endothelial growth factor, basic fibroblast growth factor

## Abstract

Angiogenesis plays an important role in the progression and development of benign prostatic hyperplasia (BPH), and has become a promising target for BPH treatment. The hypoxia-inducible factor-1α (HIF-1α) signaling pathway promotes the process of angiogenesis, contributing to the growth and progression of a number of hyperplasia diseases, including BPH. Qianliening capsule (QC) is a traditional Chinese formula that has been used clinically in China to treat BPH for a number of years. Recently, QC was demonstrated to inhibit prostatic cell growth and induce apoptosis *in vivo* and *in vitro* via regulating the epidermal growth factor/signal transducer and activator of transcription 3 signaling pathway and mitochondrion-dependent apoptosis pathway. However, the mechanisms underlying the anti-BPH effect remain largely unknown. To further elucidate the mechanism of QC activity in BPH treatment, a rat BPH model established by injecting testosterone following castration was established and the effect of QC on prostatic tissue angiogenesis was evaluated, as well as the underlying molecular mechanisms. QC was shown to reduce the prostatic index in BPH rats, but without affecting the body weight, demonstrating that QC is effective in the treatment of BPH and without apparent toxicity. In addition, QC treatment significantly reduced the intraprostatic microvessel density, indicating antiangiogenesis activity *in vivo*. In addition, treatment with QC inhibited the expression of HIF-1α in BPH rats, as well as the expression of vascular endothelial growth factor and basic fibroblast growth factor. Therefore, for the first time, the present study hypothesized that QC inhibits angiogenesis in prostatic tissue of BPH rats via the inhibition of the HIF-1α signaling pathway, which may be one of the mechanisms in which QC treats BPH.

## Introduction

Benign prostatic hyperplasia (BPH), also known as benign enlargement of the prostate or adenofibromyomatous hyperplasia, is a noncancerous enlargement of the prostate gland, which increases linearly with aging and has become a generally observed major disease among older males (1,2). An estimated 50% of males show histological evidence of BPH by the age of 50 years and 80% by the age of 70 years (2). BPH involves hyperplasia of prostatic stromal and epithelial cells, resulting in the formation of large, fairly discrete nodules in the periurethral region of the prostate. The enlarged prostate gland interferes with the normal flow of urine. BPH leads to lower urinary tract symptoms (LUTS), including urinary hesitancy, frequent urination, urgency, thin urine flow and urinary retention (3,4). These symptoms greatly affect the physical and mental health of patients, as well as their living quality. Delayed treatment is likely to cause a number of severe complications, including bleeding from the prostate, recurrent infections, renal stones and even kidney failure.

The pathogenic mechanisms underlying BPH development are complex and are not yet completely understood. However, accumulating evidence indicates that the development and progression of BPH relies on angiogenesis to obtain adequate oxygen and nutrients from the nearby blood vessels by simple passive diffusion (5–9), which promotes prostatic cell proliferation and inhibits apoptosis. Angiogenesis is a physiological process involving the growth of new blood vessels from endothelial cell precursors or from the pre-existing vasculature (10,11), and is recognized to play an important role in various human disease processes, including wound healing, reproduction, embryonic development and acute and chronic inflammation (11–13). Increasing evidence has demonstrated that angiogenesis is a highly sophisticated and coordinated process. Hypoxia-inducible factor 1 (HIF-1) is hypothesized to be a key signaling factor required for the activation of the ‘angiogenic switch’ and the resulting increase in the expression of various angiogenic growth factors, including vascular endothelial growth factor (VEGF) and basic fibroblast growth factors (bFGF) (14–17). HIF-1 is a heterodimeric transcription factor composed of two polypeptides: HIF-1α and HIF-1β (17). HIF-1α functions as a master regulator of oxygen homeostasis and accumulates in the cytosol. Following translocation to the nucleus, it activates hypoxia-sensitive genes and regulates genes encoding angiogenic cytokines, including VEGF and bFGF. VEGF or bFGF primarily bind to the specific receptors located on vascular endothelial cells (ECs), which induces EC proliferation, migration, survival, sprouting and eventually extending the primitive vascular network (18–21). Thus, inhibition of angiogenesis may be a key target for BPH treatment.

To date, pharmacotherapy remains the modality of choice for BPH treatment, and can be roughly divided into three groups: 5α-reductase inhibitors, α-adrenergic blockers and alternative therapies. The 5α-reductase inhibitors, including finasteride and dutasteride, inhibit dihydrotestosterone (DHT) production by suppressing 5α-reductase. The α-adrenergic blockers, including terazosin, doxazosin and tamsulosin, inhibit α-adrenergic receptors (22,23), which relaxes smooth muscle in the prostate and bladder neck, resulting in a decreased blockage of urine flow. However, these prescription medications can have troubling side effects, including orthostatic hypotension, decreased libido and ejaculation or erectile dysfunction (24–27). Due to these side effects, natural products that appear to have limited adverse events are becoming increasingly important in the treatment of BPH, including saw palmetto, pygeum africanum and hypoxis rooperi (28–30), which have long been used to treat BPH successfully.

Qianliening capsule (QC) is a traditional Chinese medicine (TCM) formulation that consists of a combination of rhubarb, leech, astragalus, achyranthes and dodder. Previous studies have shown that QC exhibits properties of heat-clearing, detoxification, promotion of blood circulation and removal of blood stasis, as well as tonifying the kidney and nourishing vitality (replenishing the kidney qi in Chinese medicine) (31,32). QC has been reported to exhibit significant therapeutic effects in BPH by markedly improving a series of LUTS and the dynamic index of urine flow in BPH patients (31). In addition, accumulating evidence has shown that QC markedly decreases the prostatic volume and weight, inhibits prostatic hyperplasia, relieves the abnormal ratio of estrogen to androgen, regulates the expression of the estrogen and androgen receptors, induces prostatic cell apoptosis and suppresses cell proliferation by regulating the epidermal growth factor/signal transducer and activator of transcription 3 signaling pathway and mitochondrion-dependent apoptosis pathway (32–36). However, the precise mechanism underlying the anti-BPH activity remains largely unclear. To further elucidate the mechanism of QC activity in BPH, the present study evaluated the effect of QC extracts on angiogenesis in a rat model of BPH, and investigated the underlying molecular mechanisms.

## Materials and methods

### Drugs and reagents

QC (FDA approval no. Z20110009; Fujian, China), as previously described (35), was provided by the Academy of Pharmacology of Fujian University of Traditional Chinese Medicine (Fuzhou, China). The drug powder inside the QC was dissolved in distilled water and stored at 4°C. Testosterone propionate injection solution (25 mg/m1) was obtained from Shanghai GM Pharmaceutical Co., Ltd (Shanghai, China) and TRIzol reagent was purchased from Invitrogen Life Technologies (Carlsbad, CA, USA). SuperScript II reverse transcriptase was obtained from Promega Corporation (Madison, WI, USA). Rat VEGF and bFGF ELISA kits were obtained from Shanghai XiTang Biological Technology, Co., Ltd. (Shanghai, China). All antibodies were purchased from Hebei Bohai Biotechnology Development Co., Ltd. (Shijiazhuang, China) and all other chemicals, unless otherwise stated, were obtained from Sigma-Adrich (St. Louis, MO, USA).

### Animals

In total, 30 specific-pathogen free male Sprague-Dawley (SD) rats (initial body weight, 200–220 g) were purchased from Shanghai Si-Lai-Ke Experimental Animal, Co., Ltd. (Shanghai, China). Rats were housed in clean pathogen-free rooms in an environment with controlled temperature (22°C), humidity and a 12 h light/dark cycle with free access to water and a standard laboratory diet. All animal treatments were performed. The study was conducted in accordance with International Ethical guidelines and the National Institutes of Health Guide concerning the Care and Use of Laboratory Animals. Experimental protocols were approved by the Institutional Animal Care and Use Committee of Fujian University of Traditional Chinese Medicine.

### Grouping and establishing a rat BPH model and drug administration

A rat model of BPH was generated as previously described (35). Briefly, the rat model of BPH was induced by subcutaneous injection of testosterone propionate following castration. The scrota of 20 rats, out of a total 30 male SD rats, were castrated. One week after surgery, the rats were randomly divided into three groups (n=10), termed the control (10 ml/kg saline), model (10 ml/kg saline) and QC groups, in which the rats were orally treated with 4.5 g/kg QC. Rats in the treated groups received the corresponding drug dosage by gastrogavage, together with subcutaneous injection of testosterone propionate (5 mg/kg) daily for 28 days.

### Sample collection

At the end of the treatments, the animals were weighed and anesthetized with ketamine-diazepam via intraperitoneal injection. Blood samples were collected aseptically from the abdominal aorta and blood-containing tubes were allowed to stand at room temperature for 2 h. Serum was obtained by centrifugation at 2,000 × g for 20 min at 4°C, and was then stored at −80°C. Intact prostate tissue was dissociated and removed with caution. The prostate index (PI) was calculated as follows: Prostate weight (PW)/body weight (BW) × 100%. One section of prostate tissue was collected from the same position and fixed with 10% formalin or stored in liquid nitrogen for later analyses.

### Detection of VEGF and bFGF serum levels by ELISA

ELISA analysis was performed as previously described (10,35). Serum levels of VEGF and bFGF were measured using ELISA kits, according to the manufacturer’s instructions. The concentrations of VEGF and bFGF were determined by comparison with serial dilutions of VEGF and bFGF purified standards.

### RNA extraction and reverse transcription-polymerase chain reaction (RT-PCR) analysis

Total RNA was isolated from fresh prostate tissues using TRIzol reagent. Oligo(dT)-primed RNA (1 μg) was reverse-transcribed with SuperScript II reverse transcriptase (Promega), according to the manufacturer’s instructions. The obtained cDNA was used to determine the mRNA expression levels of VEGF and bFGF by PCR with *Taq* DNA polymerase (Fermentas; Thermo Fisher Scientific, Waltham, MA, USA), where β-actin was used as an internal control. The sequences of the primers used for the amplification of the VEGF, bFGF and β-actin transcripts were as follows: VEGF forward, 5′-CAT CCT GGC CTC GCT GTC-3′ and reverse, 5′-CTC GCT CCA ACC GAC TGC-3′ (melting temperature, 61°C; length, 345 bp); bFGF forward, 5′-GCA TGC CCG CAC TGC CGG AGG A-3′ and reverse, 5′-GCT CAG CTC TTA GCA GAC-3′ (melting temperature, 60°C; length, 420 bp); β-actin forward, 5′-ACT GGC ATT GTG ATG GAC TC-3′ and reverse, 5′-CAG CAC TGT GTT GGC ATA GA-3′ (melting temperature, 55°C; length, 453 bp). Samples were analyzed by gel electrophoresis (1.5% agarose) and the DNA bands were examined using a Gel Documentation System (Model Gel Doc 2000; Bio-Rad, Hercules, CA, USA).

### Immunohistochemical analysis

Tissues were fixed in 10% formaldehyde for 12 h, paraffin-embedded, sectioned and placed on slides. The slides were subjected to antigen retrieval and endogenous peroxidase activity was quenched with hydrogen peroxide. Nonspecific binding was blocked with normal serum in phosphate-buffered saline (PBS; 0.1% Tween-20). Polyclonal rabbit anti-rat antibodies against CD31, HIF-1α, VEGF and bFGF (all at 1:200 dilution) were used to detect the relevant proteins. Binding of the primary antibody was demonstrated with a biotinylated secondary horseradish peroxidase-conjugated streptavidin antibody (Dako UK Ltd, Cambridge, UK) and diamino-benzidine as the chromogen. The tissues were counterstained with diluted Harris hematoxylin. Following staining, five high-power fields (magnification, ×400) were randomly selected in each slide. The proportion of positive cells in each field was determined using a true color multifunctional cell image analysis management system (Image-Pro Plus; Media Cybernetics, Rockville, MD, USA). To account for nonspecific staining, PBS was used to replace the primary antibody as a negative control.

### Statistical analysis

Data are presented as the mean ± standard deviation for the indicated number of independently performed experiments. Data were analyzed using the SPSS package for Windows (version 17.0; SPSS, Inc., Chicago, IL, USA). Statistical analyses were conducted with the Student’s t-test and analysis of variance, where P<0.05 was considered to indicate a statistically significant difference.

## Results

### Effects of QC on the BW and PI

**Figure 1:**
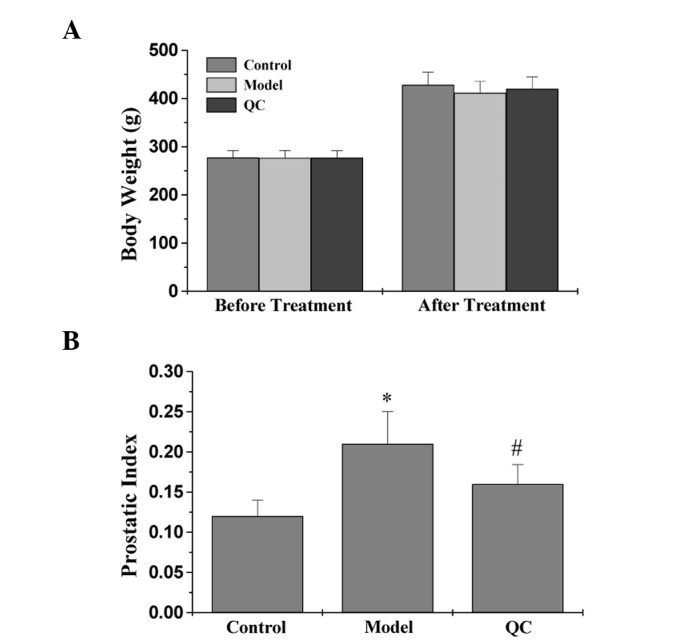
Effect of QC treatment on the (A) BW and (B) PI. Data are expressed as the mean ± standard deviation (error bars) from 10 individual rats in each group. ^*^P<0.01, vs. control; ^#^P<0.05, vs. model. BW, body weight; PI, prostatic index; QC, qianliening capsule.

Whether QC treatment caused any adverse health effects during the study was monitored by measuring BW gain. This is a relevant and widely used primary indicator to assess the gross toxicity of testing drugs in intervention studies. As shown in Fig. 1A, oral administration of QC did not affect the BW gain and was almost comparable with the respective control groups (P>0.05), which was consistent with a previous study of toxicity (37). To evaluate the efficacy of QC in the treatment of BPH, the effect of QC on the PI was assessed in BPH rats by calculating the ratio of PW to BW. In the model group, the PI increased significantly compared with the control group (P<0.01; Fig. 1B), which continued for a period of 28 days, indicating successful model construction. However, treatment with QC significantly reduced the PI in the BPH rats when compared with the model group (P<0.01; Fig. 1B). These observations indicated that QC exhibits efficacy for the treatment of BPH in rats, without any apparent signs of toxicity.

### QC exhibits antiangiogenic activity in prostatic tissue of BPH rats

**Figure 2:**
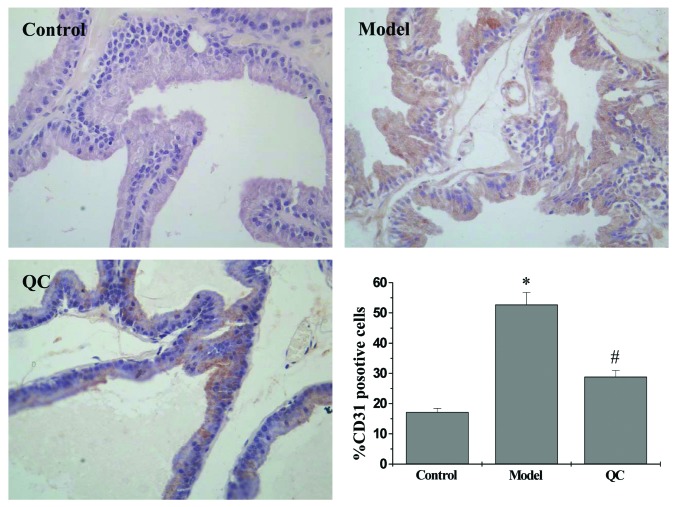
Inhibition effect of QC on the intraprostatic MVD in BPH rats. CD31 expression was observed using IHC staining (magnification, ×400) and quantification of the IHC assay was represented as the percentage of positively-stained cells. Data are expressed as the mean ± standard deviation (error bars) from 10 individual rats in each group. ^*^P<0.01, vs. control; ^#^P<0.01, vs. model. QC, qianliening capsule; BPH, benign prostatic hyperplasia; IHC, immunohistochemistry; MVD, microvessel density.

IHC staining for the EC-specific marker, CD31, was performed to determine the effect of QC on intratumoral microvessel density (MVD), which is considered to be an indicator of angiogenesis. As shown in Fig. 2, the percentage of CD31-positive cells in the model group significantly increased when compared with the control group (P<0.01); however, treatment with QC significantly decreased the number of CD31-positive cells when compared with the model group. These observations indicated that inhibition of BPH tissue angiogenesis by QC may contribute to the inhibition of prostatic cell proliferation.

### QC downregulates the expression of VEGF and bFGF in BPH rats

**Figure 3:**
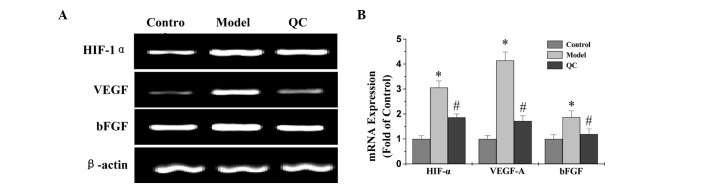
Effect of QC treatment on the mRNA expression levels of HIF-1α, VEGF and bFGF in prostatic tissue. (A) mRNA expression levels of HIF-1α, VEGF and bFGF in prostatic tissue were determined by qPCR and shown by electrophoresis, with β-actin as an internal control. (B) Densitometric analysis data were normalized against the mean mRNA expression of the control group (100%). ^*^P<0.01, vs. control; ^#^P<0.01, vs. model. QC, qianliening capsule, HIF-1α, hypoxia-inducible factor-1α; VEGF, vascular endothelial growth factor; bFGF, basic fibroblast growth factor; qPCR, quantitative polymerase chain reaction.

**Figure 4:**
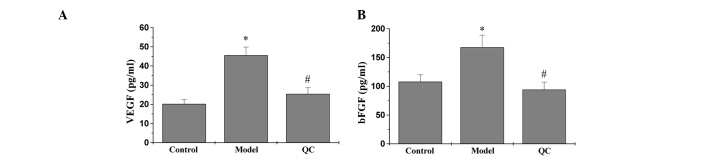
Effect of QC treatment on the secretion levels of (A) VEGF and (B) bFGF in the serum, as determined by ELISA. Data are expressed as the mean ± standard deviation (error bars) from 10 individual rats in each group. ^*^P<0.01, vs. control; ^#^P<0.01, vs. model. QC, qianliening capsule; VEGF, vascular endothelial growth factor; bFGF, basic fibroblast growth factor.

**Figure 5:**
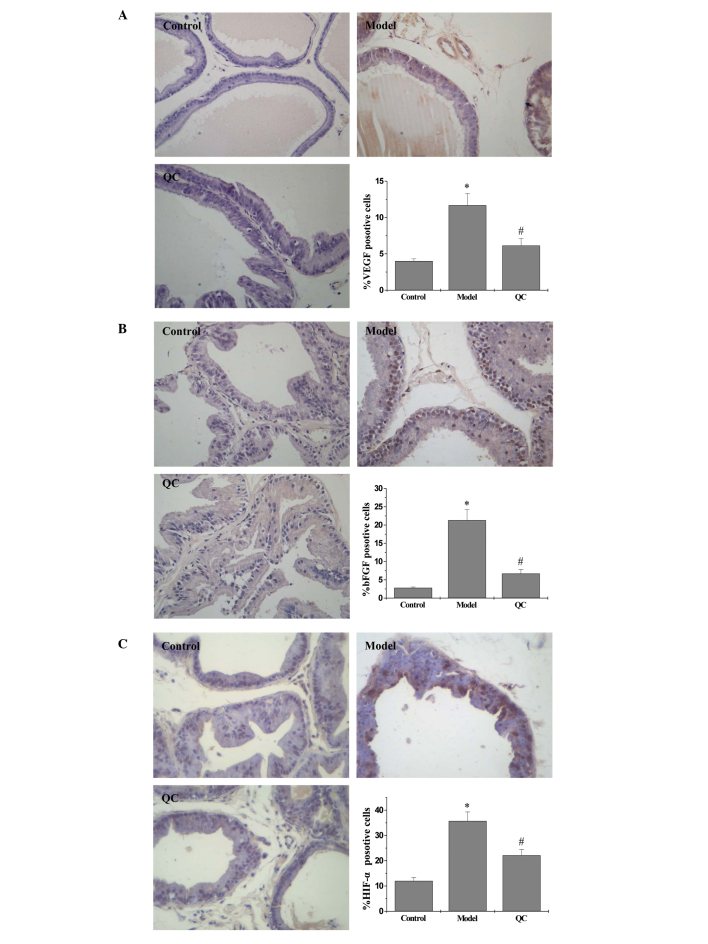
Effect of QC treatment on the protein expression of (A) VEGF, (B) bFGF and (C) HIF-1α in prostatic tissue (IHC staining; magnification, ×400). The mean proportion of positive cells in each field were counted using a true color multifunctional cell image analysis management system. Data are expressed as the mean ± standard deviation (error bars) from 10 individual rats in each group. ^*^P<0.01, vs. control; ^#^P<0.01, vs. model. QC, qianliening capsule; HIF-1α, hypoxia-inducible factor-1α; VEGF, vascular endothelial growth factor; bFGF, basic fibroblast growth factor; IHC, immunohistochemistry.

Protein and mRNA expression levels of VEGF and bFGF in the prostatic tissue of BPH rats were detected using IHC and qPCR analysis, respectively, while the secretion levels of VEGF and bFGF in the serum were analyzed by ELISA. The results of the qPCR assay revealed that the mRNA expression levels of VEGF and bFGF in the model group were significantly increased when compared with the control group (P<0.01). However, a marked decrease in expression was observed following treatment with QC when compared with the model group (Fig. 3). ELISA and IHC observations showed that the protein expression patterns of VEGF and bFGF were similar to the respective mRNA expression levels (Figs. 4, 5A and B).

### QC suppresses the HIF-1α signaling pathway in BPH rats

To determine the effect of QC on the HIF-1α signaling pathway, the mRNA and protein expression levels of HIF-1α were evaluated in the prostatic tissue of BPH rats using qPCR and IHC assays. The qPCR results showed that the mRNA expression levels of HIF-1α in the model group were significantly increased when compared with the normal group (P<0.01); however, expression was downregulated following treatment with QC (Fig. 3). Protein expression levels of HIF-1α in the prostatic tissue of BPH rats were determined using IHC. As shown in Fig. 5C, the positive expression levels of HIF-1α in the model group were markedly increased compared with the normal group (P<0.01); however, treatment with QC significantly inhibited the effect that the model construction had on HIF-1α expression. Therefore, these observations indicated that QC inhibits HIF-1α signaling in BPH rats.

## Discussion

BPH is the most common proliferative disease of the prostate in males, greater than 90% of men are affected by the age of 80. The pathogenesis of BPH is complex and not yet elucidated. Major theories of BPH pathogenesis include DHT, interstitium-epithelial cell interaction, androgen-estrogen coordination, embryo rearousal, cell proliferation and apoptosis disorder (38,39). Recently, angiogenesis has become an attractive target for BPH treatment due to its essential role in the progression and development of BPH. Angiogenesis is a complex process and regulated by a variety of molecules. Therefore, BPH treatment reagents that affect a single target may be insufficient and long-term administration may generate side effects. These problems highlight the urgency for the development of multi-target agents with minimal side effects and toxicity. Natural products, including TCMs, have been used clinically to treat various types of diseases, including BPH (28,29,40). QC, a TCM formulation, is a complex combination of numerous natural products, each of which contains a number of chemical compounds. Therefore, QC is considered to be a multi-component and multi-target agent that exerts a therapeutic function in a more holistic manner; and is a promising approach for BPH treatment. However, the mechanism underlying the anti-BPH activity remains largely unknown.

In the present study, QC was confirmed to significantly reduce the PI in BPH rats without affecting the BW, consistent with the observations of a previous study (35). Notably, through IHC staining for the endothelial cell-specific marker, CD31, QC was found to significantly reduce the intraprostatic MVD, demonstrating an *in vivo* antiangiogenic activity. Angiogenesis is tightly regulated by the HIF-1α signaling pathway, since activation of HIF-1α signaling upregulates the expression of VEGF and bFGF, which are strong angiogenesis stimulators. VEGF and bFGF exert a proangiogenic function via binding to specific receptors, leading to a series of angiogenic processes (18,41). In the present study, QC treatment was shown to inhibit the activation of the HIF-1α pathway in prostatic hyperplasia tissues, with QC significantly suppressing the mRNA and protein expression of HIF-1α. Consistently, administration of QC significantly decreased the serum levels of VEGF and bFGF in BPH rats, as well as downregulated the mRNA and protein expression levels of VEGF and bFGF in prostatic tissue.

Angiogenesis is regulated by multiple pathways, including the Ras/extracellular signal-regulated kinase, phosphatidylinositol 3-kinase/Akt/mammalian target of rapamycin, Hedgehog and Wnt signaling pathways. In addition, QC is composed of rhubarb, leech, astragalus, achyranthes and dodder, which contain a number of chemical compounds, including emodin, calycosin, hirudin, D-β-asparagine, astragalan, rhein, chrysophanol, oleanolic acid, ursolic acid, ecdysterone, isocyasterone, quercetin, coumarin and stigmasterol. It is unknown which of these compounds contribute to the anti-BPH activity, or whether the compounds of QC target various sites individually or function on a single site additively or synergistically. It also remains unclear which signaling pathways these compounds are involved in to exert their bioactivity. Future study should focus on addressing these questions in order to completely elucidate the molecular mechanism by which QC treats BPH, with the aim of developing improved multi-target drugs for BPH therapy.

In conclusion, for the first time, the present study has demonstrated that QC inhibits angiogenesis in prostatic tissue of BPH rats via the inhibition of the HIF-1α signaling pathway, which may in part explain the mechanism underlying the activity of QC in BPH treatment.

## Abbreviations

QC: qianliening capsule
BPH: benign prostatic hyperplasia
HIF-1α: hypoxia-inducible factor-1α
VEGF: vascular endothelial growth factor
bFGF: basic fibroblast growth factor
BW: body weight
PI: prostatic index
LUTS: lower urinary tract symptoms
EC: endothelial cell
DHT: dihydrotestosterone
SD: Sprague-Dawley
PW: prostate weight
RT-PCR: reverse transcription- polymerase chain reaction
PBS: phosphate-buffered saline
MVD: microvessel density
TCM: traditional Chinese medicine
